# A Novel Glycerol Kinase Gene *OsNHO1* Regulates Resistance to Bacterial Blight and Blast Diseases in Rice

**DOI:** 10.3389/fpls.2021.800625

**Published:** 2022-01-20

**Authors:** Xiaorong Xiao, Rui Wang, Shahneela Khaskhali, Zhiliang Gao, Wenya Guo, Honggang Wang, Xiaolei Niu, Chaoze He, Xiaohui Yu, Yinhua Chen

**Affiliations:** ^1^Hainan Key Laboratory for Sustainable Utilization of Tropical Bioresources, College of Tropical Crops, Hainan University, Haikou, China; ^2^School of Life Science, Hainan University, Haikou, China; ^3^Cereal Crops Institute, Hainan Academy of Agricultural Sciences/Sanya Institute, Hainan Academy of Agricultural Sciences, Sanya, China

**Keywords:** rice, glycerol kinase, non-host resistance, bacterial blight, pathogen, wax

## Abstract

Glycerol-induced resistance to various pathogens has been reported in different plants. Glycerol kinase (GK), a vital rate-limiting enzyme that catalyzes glycerol conversion to glycerol-3-phosphate (G3P), participates in responses to both abiotic and biotic stresses. However, its physiological importance in rice defenses against pathogens remains unclear. In this research, quantification analysis revealed that GK levels were significantly induced in rice leaves infected by *Xanthomonas oryzae* pv. *oryzae (Xoo)* strain PXO99. A typical GK-encoding gene *OsNHO1* was cloned in rice. The transcriptional levels of *OsNHO1* were significantly induced by salicylic acid, jasmonic acid, and *Xoo-*PXO99. Ectopic expression of *OsNHO1* partially rescued the resistance to *P. s. pv. phaseolicola* in the *Arabidopsis nho1* mutant. In the overexpressing transgenic rice lines (*OsNHO1*-OE), the content of GK and the transcriptional level of *OsNHO1* were increased and the resistance to bacterial blight and blast was improved, while reduced *OsNHO1* expression impaired the resistance in *OsNHO1*-RNAi lines. The wax contents and expression of the wax synthesis regulatory genes were significantly increased in the overexpression lines but decreased in the *OsNHO1*-RNAi lines. We then confirmed the interaction partner of OsNHO1 using yeast two-hybrid and bimolecular fluorescence complementation assays. The transcription of the interaction partner-encoding genes *OsSRC2* and *OsPRs* in *OsNHO1*-RNAi lines was downregulated but upregulated in *OsNHO1*-OE lines. Thus, we concluded that *OsNHO1* provided disease resistance by affecting the wax content and modulating the transcription levels of *PR* genes.

## Introduction

Plants are threatened by many pathogens through environmental exposure. However, they have evolved a series of complex defense mechanisms. Constitutive resistance and inducible resistance are two typical defense mechanisms by which plants deal with pathogen invasion (Mysore and Ryu, 2004; Ellis, 2006). The constitutive defenses are provided by plant cell walls, cytoskeleton, obstacles, and a variety of secondary metabolites (Yun et al., 2003). Induced defenses include the accumulation of active antimicrobial substances, the activation of plant defense signal transduction pathways, calcium influx, the accumulation of reactive oxygen species (ROS), the production of nitric oxide, the occurrence of hypersensitivity reactions, the expression of defense-related genes, etc. (Lipka, 2005; Lee et al., 2017). In recent decades, significant progress has been made in understanding inducible defense mechanisms, ranging from the pathogen-associated molecular pattern (PAMP)-induced basal resistance to effector-induced cultivar-specific resistance (Jones and Dangl, 2006; Delventhal et al., 2017).

Recent evidence has suggested that primary metabolic pathways and metabolic signaling in both plants and pathogens can interface with disease-related signaling (Rolland and Sheen, 2002). The components of primary metabolism can act as signals regulating plant defense (Schaaf and Hess, 1995; Chandra-Shekara et al., 2007). Both the fatty acid and carbohydrate metabolism play important roles in plant defense and are involved in cross-talk with various phytohormones, namely, salicylic acid (SA), jasmonic acid (JA), and abscisic acid (ABA) (Scheideler et al., 2002; Kachroo et al., 2003, 2004, 2005). Vitamin B1 and sucrose also induce resistance to pathogens in *Arabidopsis* and rice (*Oryza sativa*), respectively (Ahn et al., 2005; Gómez-Ariza et al., 2007).

Glycerol is a common cellular metabolite present in a wide range of organisms. Glycerol metabolism is initiated upon its conversion to glycerol-3-phosphate (G3P), which can be derived *via* glycerol kinase (GK)-mediated phosphorylation of glycerol or G3P dehydrogenase (G3Pdh)-mediated reduction of dihydroxyacetone phosphate (DHAP) (Chanda et al., 2008). The participation of glycerol and its metabolites in host defense has been reported in *Arabidopsis*, wheat, pepper, and soybean (Kachroo et al., 2004, 2005, 2008; Chandra-Shekara et al., 2007). *AtNHO1*, which encodes flagellin-induced GK in *Arabidopsis*, is an essential factor in gene-for-gene resistance against *Pst* DC3000 and basal resistance against *Colletotrichum higginsianum* (Kang et al., 2003; Chanda et al., 2008). G3P levels in *Arabidopsis* are associated with defense against the hemibiotrophic fungal pathogen *Colletotrichum higginsianum*. Transgenic plants that are impaired in the utilization of plastidial G3P, accumulate elevated levels of pathogen-induced G3P and display enhanced resistance. *TaGLI1*, which encodes GK, contributes to systemic acquired resistance against *Puccinia striiformis f. sp. Tritici* in wheat (Yang et al., 2013). All these previous findings suggested that regulating glycerol metabolism could enhance the immune response in plants.

Glycerol-3-phosphate can be transported between the cytosol and the plastidial stroma. In the plastids, G3P is acylated with oleic acid (18:1) by the ACT1-encoded G3P acyltransferase. This ACT1-utilized 18:1 is derived from stearoyl-acyl carrier protein (ACP)-desaturase (SSI2)-catalyzed desaturation of stearic acid (18:0). The 18:1-ACP generated by ACT1 either enters the prokaryotic lipid biosynthetic pathway through acylation of G3P or is exported from the plastids as a CoA-thioester to enter the eukaryotic lipid biosynthetic pathway (Brisson et al., 2001). C16 and C18 chain fatty acids are important precursors for wax synthesis in the endoplasmic reticulum (Wang et al., 2018). As a type of secondary metabolite, wax is widely involved in many physiological resistance processes, namely, stress defenses and resistance to pests and diseases. Wax and cutin, the main components of the cuticle, form the first line of defense against pathogen infection in plants and they play a critical role in physical resistance (physical barrier) and chemical resistance (bacteriostasis) as constitutive defense components (Ye et al., 2009). The inducible wax component can also act as a signal molecule or inducer to activate downstream resistance reactions and exert its chemical resistance function (He et al., 2018).

As a model monocot plant, rice (*Oryza sativa*) is one of the staple crops in many countries and has significant economic significance. However, the yield is adversely impacted by bacterial blight and rice blast caused by *Xanthomonas oryzae* pv. *oryzae* (*Xoo*) and *Magnaporthe oryzae* (*M. oryzae*), respectively. There is an urgent need to identify broad-spectrum resistance genes to the diseases. In this study, we isolated a rice GK gene *OsNHO1* and found that *OsNHO1* can contribute to the non-host resistance in *Arabidopsis*. Moreover, it acts as a positive regulator in resistance to *Xoo*-PXO99 and *M. oryzae* Y34. Overexpression of the *OsNHO1* gene significantly increased the wax content of transgenic plants and regulated the expression of the downstream *PR* genes. In this study, we provided evidence that modifying glycerol metabolism may also regulate the resistance of rice by affecting wax synthesis.

## Materials and Methods

### Bacterial Strains and Plants

Bacterial strains *Xanthomonas oryzae* pv. *oryzae (Xoo)-*PXO99, *M. oryzae* Y34, *P. s. phaseolicola, E. coli* DH5α, and *A. tumefaciens* were preserved and routinely cultured in our laboratory.

*Arabidopsis* ecotype Columbia [Col-0, wild type (WT)], *nho1* mutant (Col-0 background), and rice (*Oryza sativa L*.) var. *Nippobare*, DJ, and TP309 were provided by the Institute of Microbiology, the Chinese Academy of Sciences. All the *Arabidopsis* plants were grown in growth chambers at 20°C at night and 22°C during the day with a 10-h/day photoperiod. Rice plants were grown at 30°C/28°C with a 14 h/10 h day/night cycle.

### Isolation, Sequencing, and Phylogenetic Analysis of *OsNHO1*

The cDNA sequence of *OsNHO1* (LOC_Os04g55410) was obtained from the Rice Annotation Project Database (http://rice.plantbiology.msu.edu/) by BLAST with the amino acid sequence of *AtNHO1* gene (accession number: AT1G80460). We designed specific primers tailed with *Bam*H I and *Sac* I (*OsNHO1*-F/R, Supplementary Table S1) to amplify the full-length *OsNHO1*. The identification of the amplified *OsNHO1* was verified by sequencing. Multiple sequence alignment of OsNHO1 with other GK proteins was conducted using DNAMAN Version 6.0 (Lynnon Corporation, Canada). A phylogenetic tree including the OsNHO1 and other GK proteins was constructed using the MEGA7 (Tamura, Stecher, and Kumar 2016) program. The accession numbers of proteins used in multiple sequence alignment and phylogenetic analysis are listed in Supplementary Table S2.

### Cloning and Analysis of *OsNHO1* Promoter

We designed specific primers (Ppronho1-62F/R, Supplementary Table S1) to clone the promoter sequence of *OsNHO1* by chromosome walking. The fragment was sequenced, and the promoter sequence was analyzed with the PlantCARE online website (http://bioinformatics.psb.ugent.be/webtools/plantcare/html/) and visualized by TBtools software (Chen et al., 2020) (Supplementary Figure S1).

### Expression Profile Analysis of *OsNHO1*

Germinant WT (TP309) rice seedlings were grown in pots at 30°C/28°C with a 14 h/10 h day/night cycle. For hormone treatments, rice plants with 4 leaves were sprayed with 1 mmol/l SA and 0.1 mmol/l methyl jasmonate (MeJA), and the control plants were sprayed with deionized water. Leaves were sampled at 0, 1, 4, 8, 12, and 24 h after treatment. For pathogen infection, rice leaves were detached from plants and infected with *Xoo-*PXO99 (OD 0.4–0.6), and the control was infected with water. The leaves were sampled at 0, 1, 4, 8, 12, and 24 h. All the samples were immediately frozen in liquid nitrogen and stored at −80°C.

The roots, stems, and leaves of TP309 rice plants were sampled and ground in liquid nitrogen. Total RNA was extracted using the TRIzol method. DNase I (Invitrogen) was employed to digest the genomic DNA. Total RNA (1 μg) was subsequently used for first-strand cDNA synthesis catalyzed by M-MLV reverse transcriptase with Oligo d_T_18 primer. cDNA was stored at −20°C. Quantitative real-time PCR (qRT-PCR) was performed using the SYBR Green Mix PCR Kit, with *GAPDH* as an internal reference gene (Supplementary Table S1). The gene-specific primers used for qPCR analysis are given in Supplementary Table S1.

### Acquisition and Identification of Transgenic Rice Plants

The *OsNHO1* full-length fragment was digested with *Bam*H I and *Sac* I, and then inserted into the binary vector pTCK303 (Wang et al., 2004). The fragment for RNAi vector construction was amplified using specific primers (*OsNHO1*i-F/R, Supplementary Table S1) with *Spe* I/*Bam*H I and *Sac* I/*Kpn* I restriction enzyme sites. The purified fragments of approximately 600 bp were digested with *Spe* I and *Sac* I, inserted into pTCK303 in the sense orientation and then digested with *Bam*H I and *Kpn* I in the antisense orientation. The recombinant plasmids pTCK303-*NHO1* full and pTCK303-*NHO1i* were transferred into the *Agrobacterium* LBA4404 strain. Then, the two positive strains were preserved and used for rice plant transformation.

Transgenic rice plants were acquired *via* the *Agrobacterium-*mediated transformation method (Wang et al., 2004). Homozygous transgenic rice offspring were obtained after three generations of self-crossing, as detected by qRT-PCR and Western blotting.

### Pathogen Infection Experiment

*Xoo*-PXO99 was cultivated on a solid pressure-sensitive adhesive (PSA) medium at 28°C and then a single colony was grown in a liquid PSA medium at 180 revolutions per minute (rpm) for 24 h. Subsequently, the bacteria were cultivated on solid PSA medium again at 28°C in the dark for 2 d. Then, the bacterial culture was suspended in sterile water, and the solution was adjusted to OD600 = 0.5. Then, rice leaves were treated with the bacteria. Ten to twenty rice plants for each line at the four-leaf stage were included in the experiment. Three fully expanded leaves of each plant were cut for further observation and analysis. The lengths of disease speckles and diseased leaves were surveyed after 14 days.

*M. oryzae* isolate Y34 was cultivated on oatmeal agar containing 30 g/l oatmeal and 15 g/l agar. After 15 min of sterilization at 115°C, 100 μg/ml carbenicillin, 50 μg/ml kanamycin, and 50 μg/ml streptomycin were added. Rice leaves at the four-leaf stage were cut into fragments of approximately 6 cm and washed with sterile water. The leaves were placed on filter paper wetted with 100 mg/l 6-benzylaminopurine (6-BA). The two ends of each leaf were fixed with cotton to ensure that the leaf remained close to the filter paper. Bacterial colonies of the same size were selected and inoculated into each leaf using a 0.5-cm diameter perforator. The bacterial incidence in the leaves was surveyed 1 week later.

### GK and Wax Content Quantification

The content of GK in the offspring of transgenic rice was determined by the double-antibody sandwich method of ELISA, and the specific operation was carried out according to the instructions of the kit (Shanghai Jining, China, Cat.no.JN19516).

For wax quantification, we measured the wax content by the hot chloroform extraction method (Zhou et al., 2013). Leaves of WT and transgenic rice were cut into pieces and dipped into 60°C chloroform. The leaves were removed immediately after 30 s of oscillation. After the extraction volatilized at room temperature, the wax was weighed.

### Yeast Two-Hybrid Assays

To identify proteins interacting with OsNHO1, yeast transformation, and library screening were performed using the Match-Maker^TM^ GAL4 Two Hybrid System 3 (Clontech, Mountain View, California, USA). A cDNA library was generated from rice samples subjected to abiotic stress and biotic stress and cloned into pGADT7, which was used as a prey protein. The coding region of *OsNHO1* was cloned into pGBKT7 as a bait protein. The resultant vectors were used to transform yeast strain AH109. Positive clones were selected on a medium lacking leucine, threonine, and histidine, and positivity was ensured by culturing a medium lacking leucine, threonine, histidine, and adenine but containing X-α-Gal. Then, the samples were sequenced and verified using phytozome and rice annotation databases.

### Bimolecular Fluorescence Complementation Assay

For the bimolecular fluorescence complementation (BiFC) assay, the full-length coding sequences of *OsNHO1* and OsSRC2 were cloned into the binary BiFC vectors pSPYNE-35S and pSPYCE-35S using a gateway system (Walter et al., 2004). The combination vectors OsNHO1-YFPC and OsSRC2-YFPN were coinfiltrated into *Nicotiana benthamiana via* an *Agrobacterium*-mediated method. Yellow fluorescent protein (YFP) signals were detected with a confocal microscope.

### Statistical Analysis

Data are presented as the mean ± SD. Significant differences between transgenic lines and WT plants were analyzed using a *t*-test (*p* < 0.05). The displayed measured values are the means of three biological replicates.

## Results

### *OsNHO1* Encodes a Glycerol Kinase Which Can Be Induced by *Xoo*

**Figure 1:**
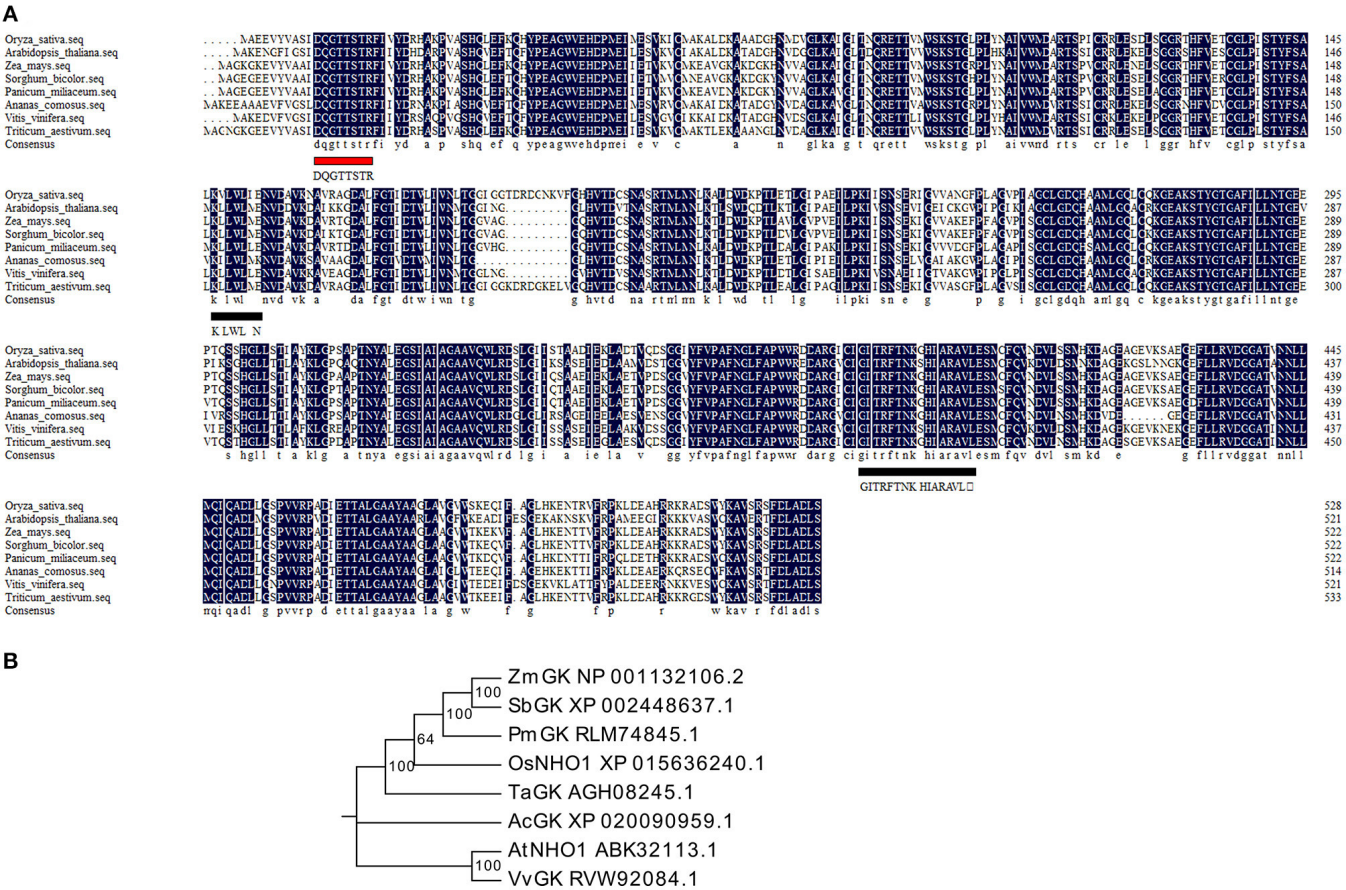
Sequence and phylogenetic analysis of OsNHO1 and GK proteins from other species. **(A)** Protein sequence alignment using DNAMAN software. The ATP-binding motif is underlined with a red solid line. The FGGY signature motives are underlined with a black solid line. **(B)** Phylogenetic analysis of GK proteins constructed using Mega6 based on the neighbor-joining method. *Oryza sativa* (accession number: XP_015636240.1), *Arabidopsis thaliana* (accession number: ABK32113.1), *Triticum aestivum* (accession number: AGH08245.1), *Zea mays* (accession number: NP_001132106.2), *Sorghum bicolor* (accession number: XP_002448637.1), *Panicum miliaceum* (accession number: RLM74845.1), *Ananas comosus* (accession number: XP_020090959.1), and *Vitis vinifera* (accession number: RVW92084.1). GK, glycerol kinase.

Glycerol kinase (GK) is the critical enzyme in the production of glycerol metabolites and belongs to the FGGY superfamily. A BLASTp search showed that only one gene locus (LOC_Os04g55410) in rice shared high homology with GKs from monocot and dicot plants. The coding sequence of this locus was cloned from rice and named the *OsNHO1* gene. Multiple sequence alignment analysis showed that OsNHO1 contained one conserved ATP-binding motif (DQGTTSTR) and two FGGY signature motifs (Figure 1A). The phylogenetic tree of GK proteins from several species showed that OsNHO1 had a close relationship with GKs from the grass family (Figure 1B). The evolutionary relationships were consistent with the relatedness of the species.

**Figure 2:**
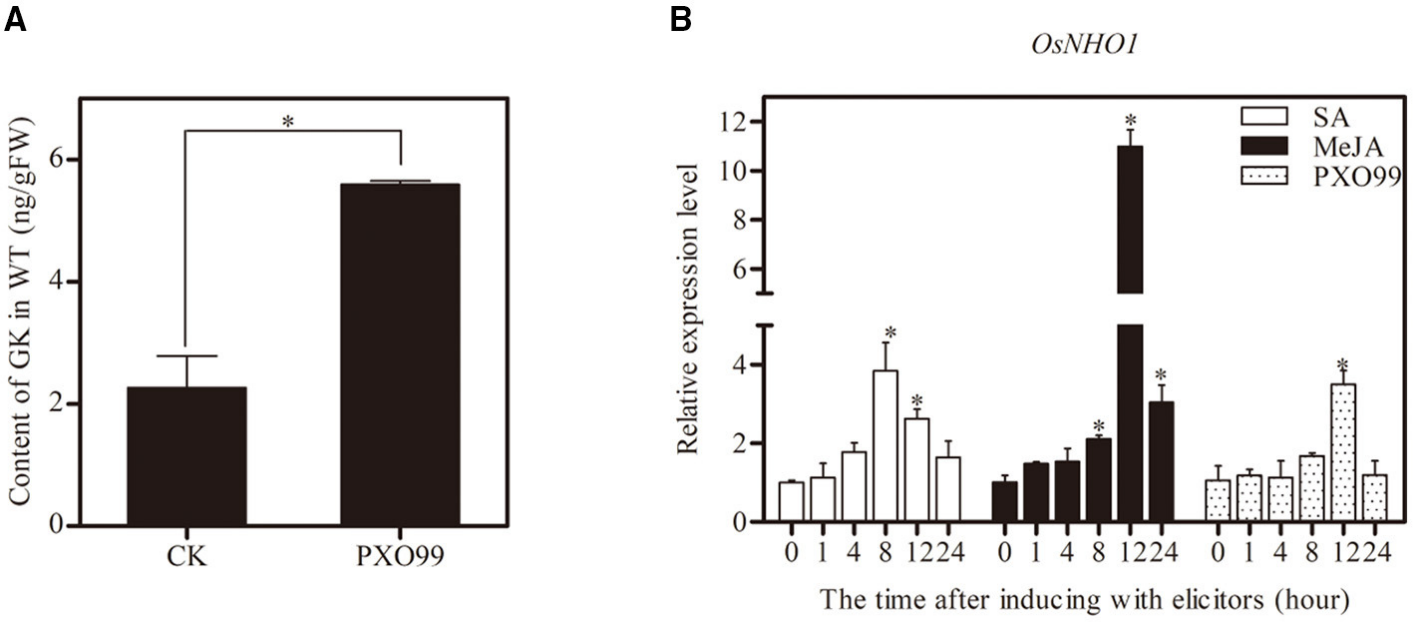
Determination of GK content and expression profiles of *OsNHO1* treated with *Xoo*-PXO99. **(A)** The GK level was increased by *Xoo* infection in rice leaves after inoculation with the *Xoo*-PXO99. **(B)** Transcriptional levels of *OsNHO1* in plants treated with JA, SA, and *Xoo*-PXO99. Each value represents the mean ± SE of three biological replicates. GK, glycerol kinase; JA, jasmonic acid; SA, salicylic acid. * indicates a significant difference at the 0.05 level.

To determine whether GK is involved in the resistance of rice against *Xoo*, we inoculated the leaves with *Xoo*-PXO99, and compared the GK content in the leaves at different time points. The GK content was strongly increased at 12 h postinoculation (hpi) compared with the control (Figure 2A). The results indicated that GK is involved in response to *Xoo*.

The expression profile of *OsNHO1* was analyzed by qPCR in rice cultivar TP309 seedlings treated with *Xoo-*PXO99, JA, and SA (Figure 2B). In the presence of JA, the relative expression of *OsNHO1* slowly increased before peaking at 12 h, with a 10-fold increase compared with that of the control. *OsNHO1* was also induced by SA. The relative expression level of *OsNHO1* increased nearly 4-fold compared with the control at 8 h and then gradually declined. When inoculated with *Xoo-*PXO99, *OsNHO1* was significantly induced only at 12 h. These results showed that *OsNHO1* could be induced by JA, SA, and *Xoo-*PXO99.

### *OsNHO1* Partially Rescued the Functional Defect of the *Arabidopsis nho1* Mutant

**Figure 3:**
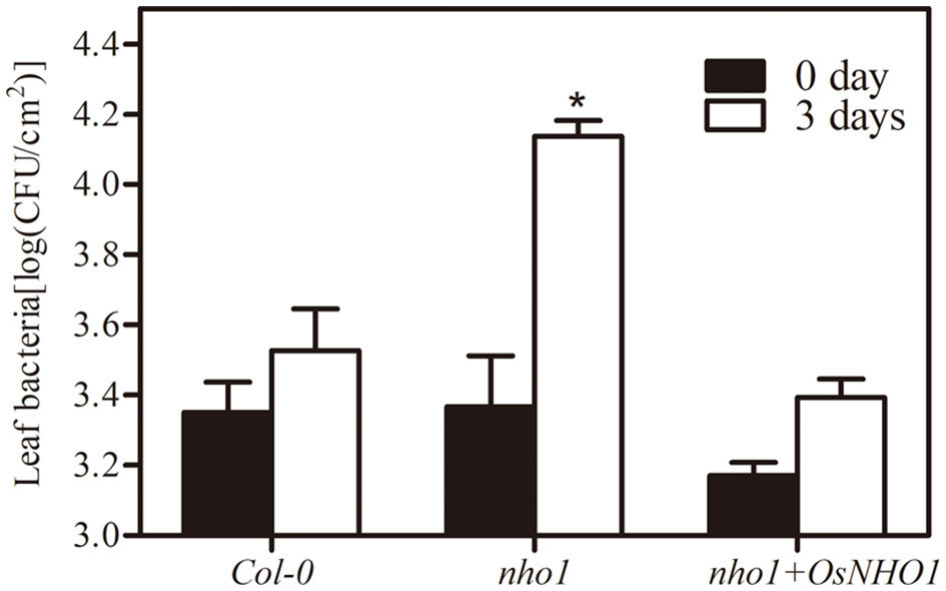
Bacterial growth of *P. s*. *phaseolicola* in Col-0, *nho1* mutant, and *nho1* + *OsNHO1* lines was measured at 0 and 3 days after inoculation. Asterisks indicate significant differences compared with the other two varieties at 3 days (*p* < 0.05) by one-way ANOVA followed by the Tukey's highest significant difference (HSD) analysis. The error bars indicate the SEs. Three independent experiments were performed with similar results.

*AtNHO1* is required for both the general and specific resistance against bacteria and fungi and is involved in flagellin-induced non-host resistance to *Pseudomonas* in *Arabidopsis*. To explore whether *OsNHO1* functions in a similar way to the homologous gene *AtNHO1* in *Arabidopsis*, we obtained *Arabidopsis nho1* mutant plants with *OsNHO1* ectopic expressed. The transgenic progeny was inoculated with the pathogen *P. s. pv. phaseolicola* and the bacterial counts were conducted at 0 d and 3 days. The bacterial number in Col-0 at 3 days was 1.6 times that at 0 day and in the *nho1* mutant, it was 5.5 times that at 0 day (Figure 3). However, in the presence of *OsNHO1*, the bacterial number at 3 days decreased to 1.8 times higher than that at 0 day. In addition, bacterial fecundity in Col-0 and *nho1* + *OsNHO1* was significantly lower than that in the *nho1* mutant (Figure 3), indicating that *OsNHO1* functions similarly to *AtNHO1* in resisting *P. s. pv. phaseolicola*.

### Regulation of *OsNHO1* Expression Could Change Defenses Against Pathogens in Transgenic Rice

**Figure 4:**
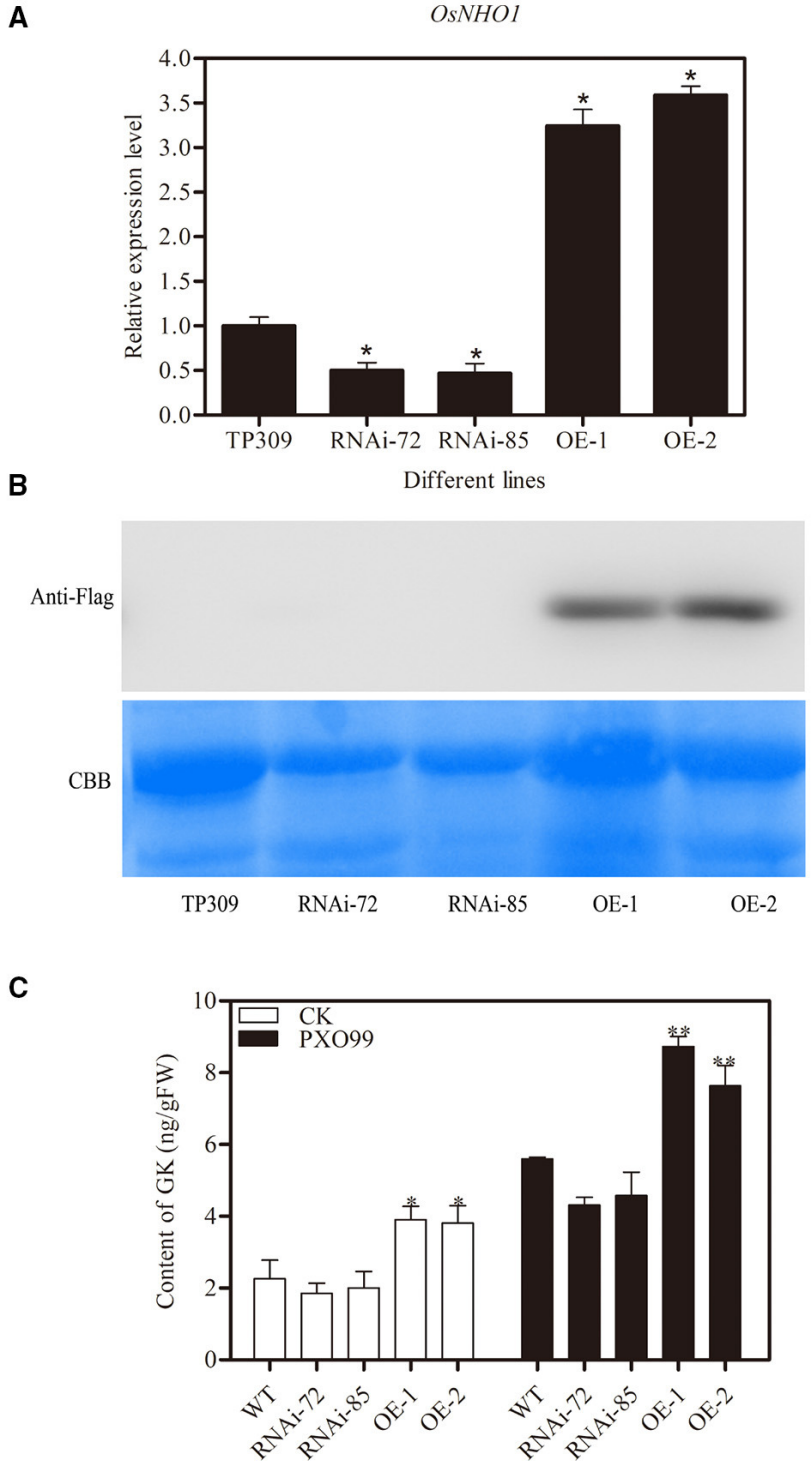
Identification of transgenic materials. **(A)** *OsNHO1* transcript levels in RNAi, OE, and WT (TP309) plants by qPCR. **(B)** Protein-level determination in different plants. **(C)** Content of GK in different transgenic plants. GK, glycerol kinase; OE, overexpression; WT, wild type. * indicates a significant difference at the 0.05 level; ** indicates a significant difference at the 0.01 level.

The *AtNHO1* was required for defense against *Pseudomonas* bacteria in *Arabidopsis* (Lu et al., 2001). To test the function of the *OsNHO1* gene in rice defense, we obtained *OsNHO1* overexpression (OE) and knockdown transgenic plants using the *Agrobacterium*-mediated genetic transformation method. After four generations of screening, homozygous transgenic lines (T4), namely, two *OsNHO1*-OE lines and two *OsNHO1*-RNAi lines, were used for subsequent research. The relative expression level of *OsNHO1* in the knockdown transgenic lines RNAi 72 and RNAi 85 was reduced by 50%, while it was increased approximately 3-fold in the OE lines *OsNHO1*-OE1 and *OsNHO1*-OE2 (Figure 4A). Western blotting results also showed that *OsNHO1* was expressed in the *OsNHO1*-OE lines (Figure 4B).

To confirm whether *OsNHO1* could affect GK accumulation, we quantified the GK contents in different transgenic plants. The content of GK in *OsNHO1*-OE plants was significantly higher than that in WT plants but was only marginally reduced in *OsNHO1*-RNAi plants compared with WT plants (Figure 4C). Meanwhile, to further verify that GK is involved in rice responses to pathogens, we measured the changes in GK contents in different materials after pathogen treatments. We found that the GK level was strongly increased in both the *OsNHO1*-OE and *OsNHO1*-RNAi lines after inoculation with *Xoo*-PXO99 at 12 hpi compared with 0 hpi (Figure 4C).

**Figure 5:**
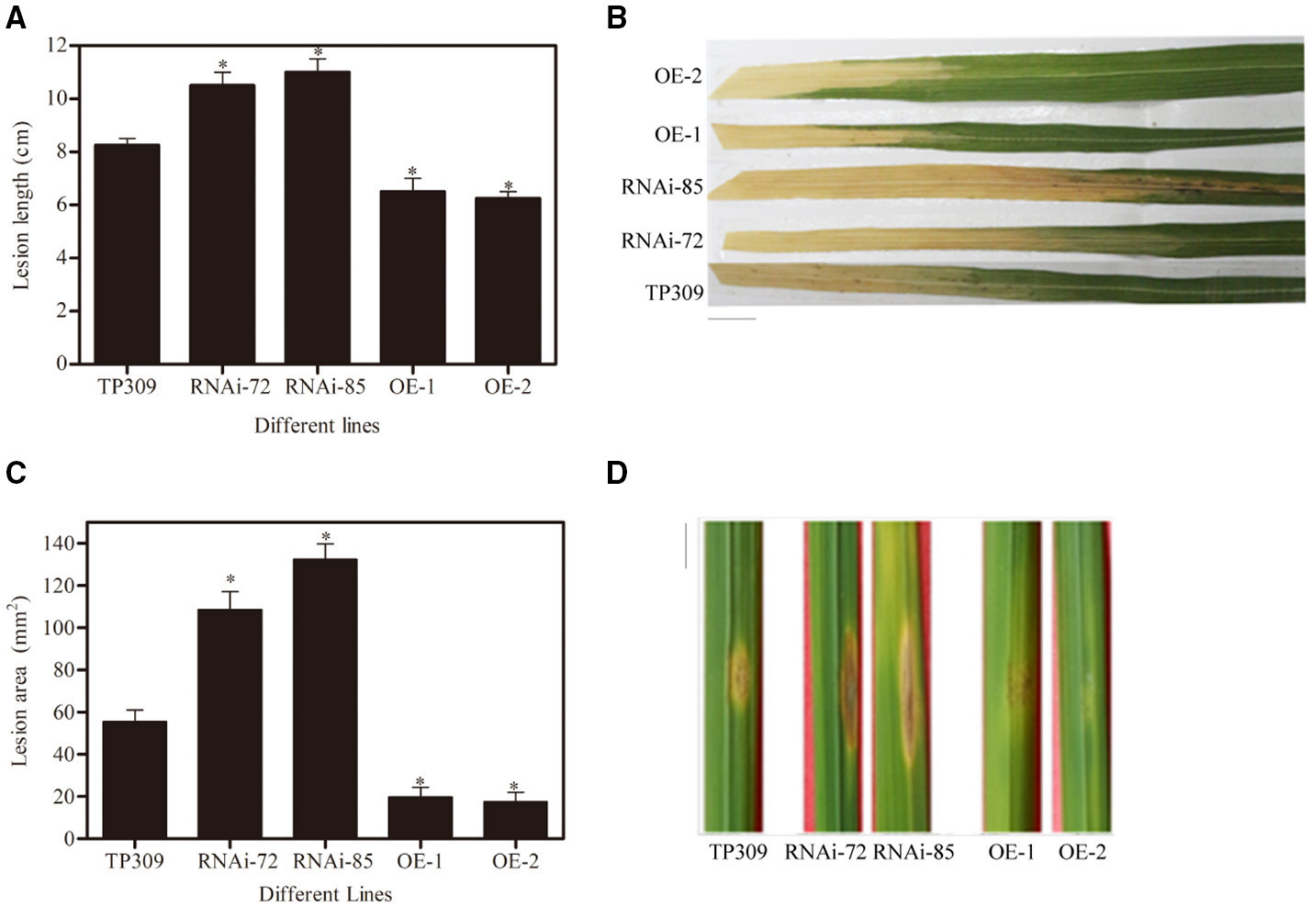
Disease resistance identification of transgenic materials. **(A)** Lesion lengths in transgenic plants were measured at 14 days postinoculation with *Xoo*-PXO99. **(B)** A photograph depicting disease symptom development in leaves 14 d postinfection with *Xoo*-PXO99. Scale bars = 1 cm. **(C)** Lesion areas of transgenic plants were measured using ImageJ software at 7 days postinoculation with Y34. **(D)** A photograph depicting disease symptom development in leaves 7 d postinfection with Y34. Scale bars = 1 cm. TP309: wild-type; RNAi-72 and RNAi-85: two RNAi-*OsNHO1* lines; OE1 and OE2, two *OsNHO1* overexpression lines. Asterisks indicate significant differences compared to TP309 (*p* < 0.05) by one-way ANOVA followed by Tukey's HSD analysis. Error bars indicate SEs. Three independent experiments were performed with similar results.

The obtained transgenic plants were used for *Xoo* and blast resistance test. We infected rice leaves with *Xoo*-PXO99 and measured the lesion length produced by *Xoo*-PXO99 after 13–15 days (Figures 5A,B), and the lesion area produced by Y34 was measured after 7–8 days (Figures 5C,D). We observed that the lesion length in the WT line was approximately 8.5 cm, while it was 11 cm in the *OsNHO1*-RNAi lines and 6 cm in the *OsNHO1*-OE lines. In addition, the lesion areas in the two overexpression lines were significantly less than those in the WT line, while the lesion areas in the two *OsNHO1*-RNAi lines were increased approximately 2-fold compared with those in the WT line.

These results showed that the defects in *OsNHO1* led to increased susceptibility to *Xoo*-PXO99 and *M. oryzae* Y34 while overexpressing *OsNHO1* inhibited the spread of bacterial blight and blast. Thus, we concluded that *OsNHO1* positively regulates rice immunity against pathogens.

### *OsNHO1* Influences the Contents of Wax

**Figure 6:**
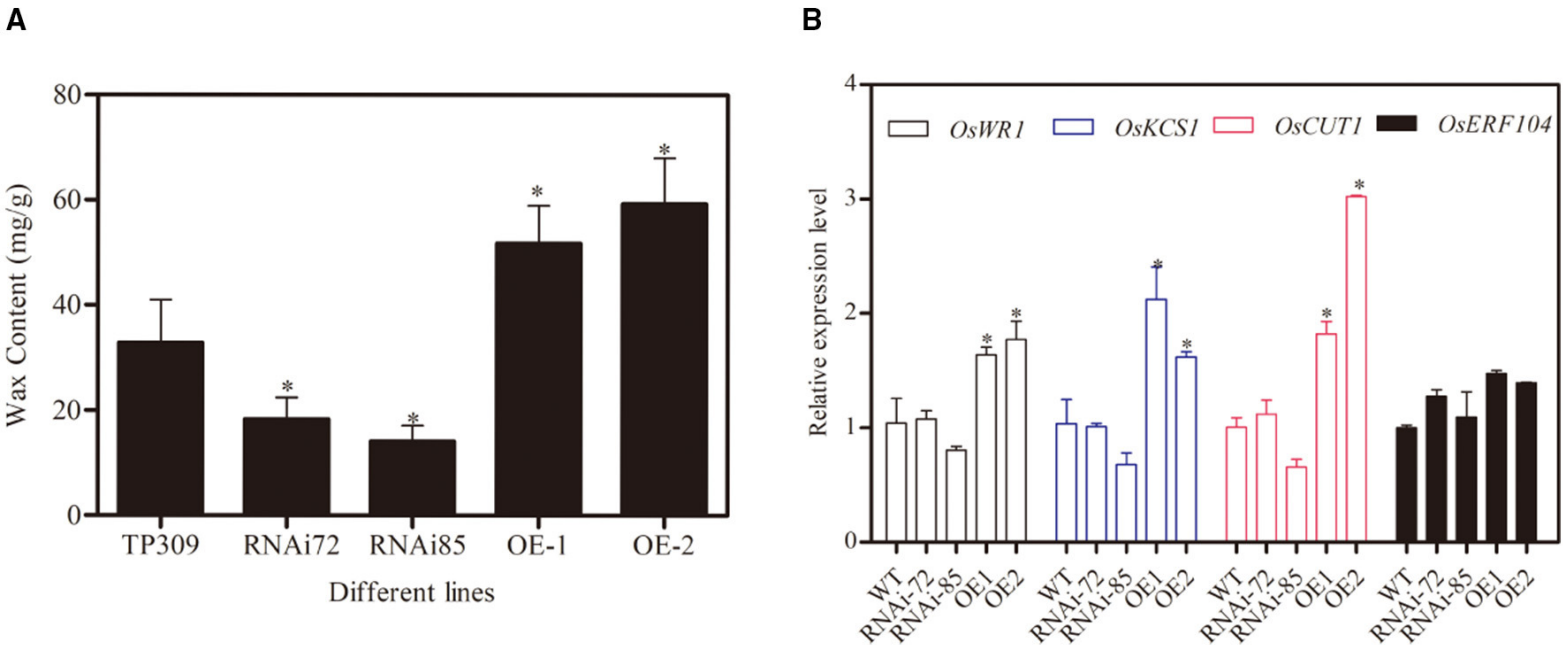
Contents of wax and relative expression of *OsWRs* genes in the WT (TP309), *OsNHO1*-RNAi, and *OsNHO1*-OE lines. **(A)** Contents of wax were measured by the hot chloroform extraction method. **(B)** Relative expression of *OsWRs* genes. Each value represents the mean ± SE of three biological replicates. Asterisks indicate significant differences compared to TP309 (*p* < 0.05) by one-way ANOVA followed by the Tukey's HSD analysis. OE, overexpression; WT, wild type.

Glycerol metabolism is initiated upon its conversion to G3P *via* the GK-mediated phosphorylation of glycerol. G3P is involved in the synthesizing of C16 and C18 chain fatty acids, which are essential precursors for wax synthesis. To analyze whether GK is related to wax synthesis, we measured the wax contents in different *OsNHO1* transgenic lines. The wax content in the *OsNHO1*-RNAi lines was reduced in comparison with that in TP309. However, the wax content in the *OsNHO1*-OE lines was higher than that in the WT (Figure 6A).

Rice wax synthesis regulatory genes (*OsWR*s) modulate wax synthesis by the alteration of long-chain fatty acids and alkanes (Wang et al., 2012; Zhou et al., 2013). In this study, we analyzed the relative transcript levels of *OsWR1, OsKCS1, OsCUT1*, and *OsERF104* in the transgenic lines (Figure 6B). These four genes were upregulated in *OsNHO1*-OE lines and showed no significant difference in the *OsNHO1*-RNAi lines compared with the WT. These results implied that overexpression of the *OsNHO1* gene increased the content of wax.

### *OsNHO1* Regulates the Expression of *OsPR5* and *OsAOS1* in Transgenic Rice Lines

**Figure 7:**
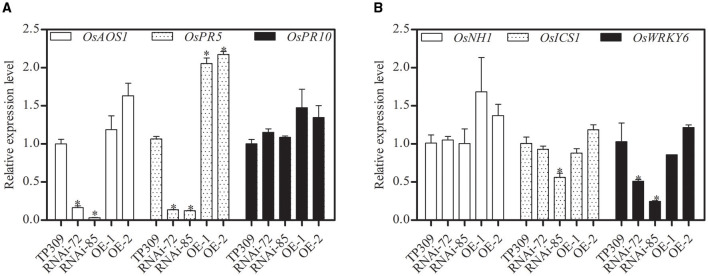
Relative expression of *OsPRs* in transgenic lines determined by qPCR with an SYBR® Premix Ex Taq TM ^II^ Kit. The transcript levels of *PR* genes in WT were considered 100%. Expression levels of the target gene were normalized to the *ACTIN* reference gene. **(A)** Relative expression of *OsAOS1, OsPR5*, and *OsPR10*. **(B)** Relative expression of *OsNH1, OsICS1*, and *OsWRKY6*. Each value represents the mean ± SE of three biological replicates. Asterisks indicate significant differences compared to TP309 (*P* < 0.05) by one-way ANOVA followed by Tukey's HSD analysis. The error bars indicate the standard errors. Three independent experiments were performed with similar results. qPCR, quantitative PCR; WT, wild type.

Glycerol-induced resistance was reported in non-host resistance with upregulation of several *PR* genes (Jiang et al., 2009; Zhang et al., 2015). Pathogenesis-related proteins (PRs) are important components in plant responses to biotic and abiotic stresses. In this study, we analyzed the relative transcript levels of *OsPRs* in the transgenic lines (Figure 7). We found that the expression of *OsPR10, OsNH1*, and *OsICS1* genes was no significant difference, while *OsPR5, OsAOS1*, and *OsWRKY6* showed different transcription levels in different materials. Silencing of *OsNHO1* decreased the relative expression of *OsPR5* by 85%, while overexpression of *OsNHO1* increased the expression of *OsPR5*.

### *OsSRC2* Is the Interaction Partner of *OsNHO1*

**Figure 8:**
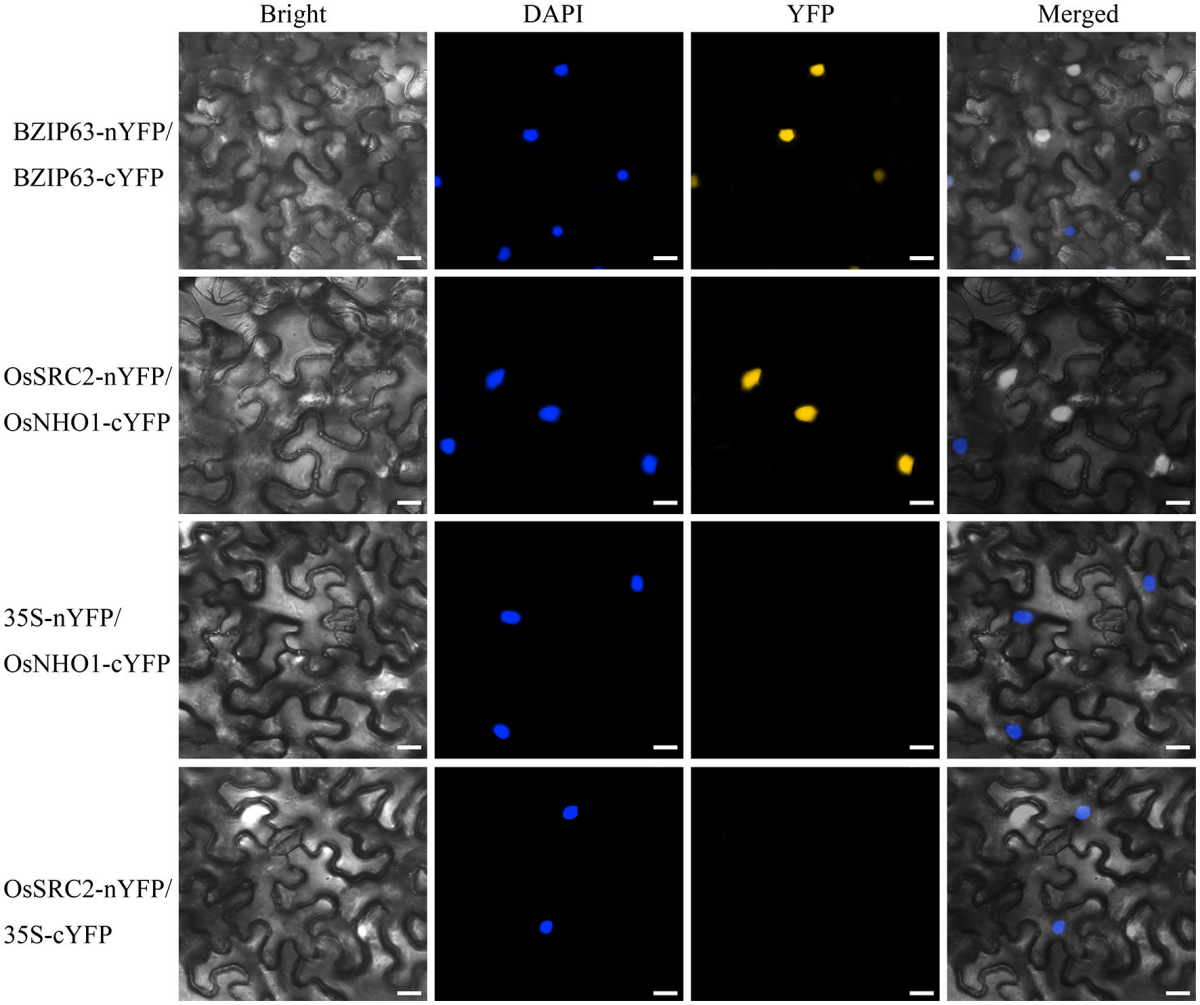
*In vivo* interaction between *OsNHO1* and OsSRC2 by BiFC analysis. *N. benthamiana* leaves were co-infiltrated with *Agrobacterium* carrying 35S::OsSRC2-nYFP and 35S::OsNHO1-cYFP. BZIP63 was used as a positive control. 35S-nYFP/OsNHO1-cYFP and OsSRC2-nYFP/35S-cYFP were used as negative controls. Fluorescence [yellow fluorescent protein (YFP)] and bright and merged field confocal images were acquired at 72 h. DAPI was used for nuclear staining. Scale bars = 20 μm.

To identify the components in the *OsNHO1*-mediated signaling pathway, we screened the interaction partners of OsNHO1 by the yeast two-hybrid method using a rice cDNA library prepared from rice leaf tissue treated with biotic stresses. We obtained 13 potential candidate proteins (Table 1). Among these candidate proteins, the protein LOC_Os01g70790 contains a C2 domain (calcium-dependent lipid-binding domain, CaLB) and exhibits highly homologous with the protein SRC2 in soybeans, which is induced by cold stress. *In vivo* interaction between OsNHO1 and OsSRC2 indicated by BiFC analysis showed that these two proteins colocalized in the nucleus (Figure 8).

**Table 1:** Partner proteins of NHO1 selected in the process of yeast two-hybrid (Y2H) screening.

**Partner protein**	**Protein ID**
OsSRC2	LOC_Os01g70790
DnaK family protein	LOC_Os01g62290
RNA recognition motif family protein	LOC_Os02g07070
PCNA-Putative DNA replicative polymerase clamp	LOC_Os02g56130
Calcium/calmodulin depedent protein kinase	LOC_Os03g17980
DnaK family protein	LOC_Os03g60620
Expressed protein	LOC_Os05g14270
MTA/SAH nucleosidase	LOC_Os06g02220
Glycosyl hydrolase, family 31	LOC_Os07g23944
Ubiquinol-cytochrome C chaperone family protein	LOC_Os07g30790
PAP fibrillin family domain containing protein	LOC_Os09g04790
C-5 cytosine-specific DNA methylase	LOC_Os10g01570
DnaK family protein	LOC_Os11g47760

C2 domain-containing proteins play important roles in plant immunity, and the OsSRC2 protein contains a C2 domain. Previous results show that OsSRC2 functions as the interacting partner of OsNHO1. To analyze whether SRC2 is regulated by NHO1, we detected the transcription levels of *OsSRC2* in different *OsNHO1* transgenic lines by qPCR (Supplementary Figure S2). *OsSRC2* was upregulated in the *OsNHO1*-OE lines but downregulated in the *OsNHO1*-RNAi lines. These results implied that OsNHO1 may interact with the OsSRC2 protein to modulate responses to pathogens.

## Discussion

### *OsNHO1* Plays an Important Role in Resisting Bacterial and Fungal Pathogens

Glycerol can be utilized as a sole carbon and energy source for both bacteria and fungi (Wei et al., 2004). This study found that glycerol may work as a significant transferred metabolite from plant to pathogen. The way to assimilate glycerol is the phosphorylative catabolic pathway, the key enzyme of glycerol metabolism is GK. Early study found that glycerol-insensitive mutants *gli1* seedings lacking glycerol kinase are more resistant to abiotic stress (Eastmond, 2004), but *NHO1* is needed for resistance to the fungal pathogen *Botrytis cinerea* and to the bacterial resistance. Consistent with results in *Arabidopsis*, we found that *OsNHO1* was significantly induced by *Xoo*-PXO99 (Figure 2). Moreover, *OsNHO1*-OE plants showed increased resistance to *Xoo*-PXO99 and Y34, and *OsNHO1*-RNAi plants were more susceptible to pathogens (Figure 5). The correlation of *OsNHO1* transcriptional levels with different reactions implies an essential role of these biochemical processes in disease resistance. A possible explanation might be that GK can alter plant glycerol pools then affect nutrient availability for pathogens.

### *OsNHO1* May Influence the Interactions Between Plants and Biotic Agents by Upregulating the Wax-Related Genes

Cutin is an extracellular lipid polymer that contributes to protective cuticle barrier functions against biotic and abiotic stresses in land plants. The cuticle wax is chemically composed of lipids. GK deficiency alters the expression of genes involved in lipid and carbohydrate metabolism (Rahib et al., 2007). In contrast, the wax biosynthetic genes *OWR1, OsKCS1*, and *OsCUT1* in *OsNHO1*-OE plants are upregulated (Figure 6B). Corresponding to waxy gene expression, the wax content in *OsNHO1*-OE materials increases accordingly (Figure 5).

In addition, cuticle wax in plants is considered to contribute to drought, insect (Lee et al., 2014), and pathogen resistance (Jenks et al., 1994; Özer et al., 2017). Studies have shown that the content of wax in plants may also be correlated with disease resistance. For example, the wax contents of maize disease-resistant varieties were significantly higher than those of susceptible varieties (Russin et al., 1997), and the wax contents of leaves of resistant cassava varieties were higher than those of susceptible varieties (Zinsou et al., 2006). Similarly, in this study, we found that the wax content in the *OsNHO1-*OE plants, which showed increased resistance to *Xoo*-PXO99 and Y34, was far greater than that in *OsNHO1-*RNAi plants (Figure 5). This indicated that abnormal *OsNHO1* expression may disturb rice pathogen resistance by altering the wax content.

Plant hormones can work as signal molecules and then affect plant responses to stress by mediating the deposition of cuticle wax. Ethylene (ETH) could increase the content and change the structure of wax to protect citrus plants from *Penicillium digitatum* invasion (Cajuste et al., 2010). Under water stress, ABA promoted the expression of several wax-synthesis genes in *Arabidopsis* by upregulating the expression of the transcription factor *MYB96* and eventually promoted the accumulation of wax (Seo et al., 2011). Exogenous SA, MeJA, and 1-aminocyclopropane-1-carboxylate (ACC) could induce the deposition of cuticle wax in *Brassica napus*. SA is essential for sugars and glycerol-mediated disease resistance (Qian et al., 2015). Glycerol-induced resistance was reported in non-host resistance with upregulation of several *PR* genes and ROS accumulation. *OsPR5* and *OsICS1* are the key genes of the SA signaling pathway. JA and cutin wax belong to the fatty acid metabolism pathway and have a common synthetic precursor (Zhang et al., 2020). *OsAOS1* is involved in the JA signal pathway. The expression of *PR* genes was significantly reduced in *Arabidopsis* waxy epidermis mutants *cer6* and *cer2* (Garbay et al., 2007), suggesting that the transcription level of *PR* genes is closely related to waxy components. Remarkably, the transcriptional level of the *OsPR5* gene in *OsNHO1*-OE plants was higher than those in WT and *OsNHO1*-RNAi plants (Figure 7A), indicating that *OsNHO1* have an additive influence on cuticular wax biosynthesis that is SA-dependent.

### *OsNHO1* Participated in a Novel Pathway Regulating Plant Defense

Glycerol metabolism is initiated upon its conversion to G3P *via* the GK-mediated phosphorylation of glycerol. G3P is involved in the synthesis of C16 and C18 chain fatty acids, which are important precursors for wax synthesis. In addition, cuticle wax in plants is considered to contribute to drought, insect (Lee et al., 2014), and pathogen resistance (Jenks et al., 1994; Özer et al., 2017). Studies have shown that the content of wax in plants may also be correlated with disease resistance. For example, the wax contents of maize disease-resistant varieties were significantly higher than those of susceptible varieties (Russin et al., 1997), and the wax contents of leaves of resistant cassava varieties were higher than those of susceptible varieties (Zinsou et al., 2006). Similarly, in our research, we found that the wax content in the *OsNHO1-*OE plants, which showed increased resistance to *Xoo*-PXO99 and Y34, was far greater than that in *OsNHO1-*RNAi plants (Figure 7). This indicated that abnormal *OsNHO1* expression may disturb rice pathogen resistance by altering the wax content.

The cuticle wax is chemically composed of lipids. In mice, SRC2 and SRC3 regulate epidermis-specific sphingolipid production (Oda et al., 2009). In plants, SRC2 is a C2 domain-containing protein or calcium-dependent lipid-binding protein. C2 domains are found in over 100 different proteins with functions ranging from signal transduction to vesicular trafficking. The C2 domain of CaSRC2-1 is crucial for plasma membrane targeting, and the PcINF1-SRC2-1 complex is required in PcINF1-induced pepper immunity (Liu et al., 2015). In addition, the transcript level of *OsSRC2* was upregulated by *M. oryzae* according to the Rice MetaSysB database (Sureshkumar et al., 2019). Other C2 domain-containing proteins, such as SS52 in pepper (Kim et al., 2008; Sakamoto et al., 2009); OsERG1, OsERG3, and GTPase-activating protein (GAP) in rice (Kim et al., 2003; Cheung et al., 2008); and BON1/CPN1, BAP1, and BAP2 in *Arabidopsis* (Liu et al., 2005; Yang et al., 2006, 2007), play important roles in plant immunity. Here, we found that the OsSCR2 protein, which is one of the interaction partners of OsNHO1 (Table 1), contains a C2 domain. BiFC results showed that these two proteins co-localized in the nucleus (Figure 7). Our results showed that the transcriptional levels of the *OsSRC2* gene in *OsNHO1*-OE plants were higher than those in WT and *OsNHO1*-RNAi plants (Supplementary Figure S2).

Thus, we concluded that *OsNHO1* participates in a novel pathway regulating plant defense. *OsNHO1* significantly contributed to pathogen resistance to bacterial blight and rice blast by interacting with OsSRC2 protein upon pathogen infection, affecting the content of wax and modulating the expression of *PR* genes. *OsNHO1* was a potential candidate gene for disease resistance engineering. Accordingly, further investigations may focus on the following important areas.

The regulation between OsNHO1 and its partner OsSRC2 should be further investigated. The roles of OsSRC2 in innate immunity and their relationships with wax generation and PR gene activation will be of great interest in future explorations.

## Data Availability Statement

The original contributions presented in the study are included in the article/Supplementary Material, further inquiries can be directed to the corresponding author/s.

## Author Contributions

YC, XY, XN, and CH designed the research. HW, WG, and ZG performed the research. XX, RW, and SK wrote the paper. All authors read and approved the final manuscript.

## Conflict of Interest

The authors declare that the research was conducted in the absence of any commercial or financial relationships that could be construed as a potential conflict of interest.

## Publisher's Note

All claims expressed in this article are solely those of the authors and do not necessarily represent those of their affiliated organizations, or those of the publisher, the editors and the reviewers. Any product that may be evaluated in this article, or claim that may be made by its manufacturer, is not guaranteed or endorsed by the publisher.
