# Supplementary material for: A Novel Glycerol Kinase Gene OsNHO1 Regulates Resistance to Bacterial Blight and Blast Diseases in Rice
Source: Front Plant Sci. 2022 Jan 20;12:800625. doi: 10.3389/fpls.2021.800625 (PMC8811351; doi:10.3389/fpls.2021.800625)
Supplement: Supplementary file 1 [file Data_Sheet_1.pdf]

Supplementary information

Table S1 **Details of primers used in this study**

Function of primers	Name of primers	Sequence of primers
To obtain the full-length CDS sequence	*OsNHO1-F*	5'- GGGGATCCATGGCGGAGGAGGTGTATGTC-3';
	OsNHO1-R	5'-CGAGCTCCTAAAGAGAAAGGTCAGCCAAATC-3'
To construct the RNAi (RI) vector	*Posnho1a-L*	5'-GGTACCACTAGTCGGAGAGAATTGGTGTGGTT-3'
	Posnho1a-R	5'-GGATCCGAGCTCTGTCTCGATGTCAGCAGGTC-3'
To identify transgenic plants	*OsHtp-1F*	5'-CTGCTCCATACAAGCCAACC-3'
	OsHtp-2R	5'-TGCCTGAAACCGAACTGC-3'
Normalization for qRT-PCR	*OsGAPDH-F*	5'-ACAGGGGAGTTGTGTTTTGC-3'
	OsGAPDH-R	5'-CCCAACCAACCACCATGATA-3'
	OsACTIN1-F	5'-GTGGTCGCCCCTCCTGAAAG-3'
	OsACTIN1-R	5'-GGCTTGCATTCTTGGGTCCG-3'
To analyze transcript level of *OsNHO1* in transgenic plants	*POsNHO1-qF*	5'-GCCTTGGAATCATTAGCACAGCAG-3'
	POsNHO1-qR	5'-CGAGCAATGTGCCCCTTGTTAGTG-3'
To analyze transcript level of *OsSRC2* in transgenic plants	SRC2-qF	5'-TCCTACCAGGTCCGCAAGAT-3'
	SRC2-qR	5'-GATTGGGTTACCTCGCCGAT-3'
To analyze transcript levels of *OsPRs* genes in transgenic plants	*OsPR5-qF*	5'-CAGTACTGCTGCACCGGCTC-3'
	OsPR5-qR	5'-ACATCGATCAGATGCCAGCTAA-3'
	OsWRKY6-qF	5'-CTCCGCAAATCACATCCAGTT-3'
	OsWRKY6-qR	5'-ACCGAGTTGTCGAAGCTGAAG-3'
	OsAOS1-qF	5'-GCTGGTGAAGAAGGACTACGA-3'
	OsAOS1-qR	5'-CCGCCGAACGAGTTGAAG-3'
	OsPR10-qF	5'-CCCTGCCGAATACGCCTAA-3'
	OsPR10-qR	5'-CTCAAACGCCACGAGAATTTG -3'
	OsNH1-qF	5'-CACGCCTAAGCCTCGGATTA -3'
	OsNH1-qR	5'-TCAGTGAGCAGCATCCTGACTAG -3'
	OsICS1-qF	5'-TATGGTGCTATCCGCTTCGAT-3'
	OsICS1-qR	5'-CGAGAACCGAGCTCTCTTCAA -3'
To analyze transcript levels of *OsWRs* genes in transgenic plants	*OsCUT1-qF*	5'-GCTGCTCTTCTTCTTCCGCCT-3'
	OsCUT1-qR	5'-GACAGGTCCAGGTTCTTCTGCT-3'
	OsKCS1-qF	5'-GAGCAGCTCCATCTGGTACGAG-3'
	OsKCS1-qR	5'-GGTAGCGGTCAATGCAATCCTC-3'
	OsERF104-qF	5'-ATGGGAGGCAACCAGGAGTA-3'
	OsERF104-qR	5'-GAGATGACATGGAGCAGCGT-3'
	OsWR1-qF	5'-AGAAGTCCCACATTGGCGTGT-3'
	OsWR1-qR	5'-GCTCAGCAACTCCTCGATCATT-3'

Table S2 **Information of amino acid used in this study**

>OsNHO1 XP_015636240.1
MAEEVYVASIDQGTTSTRFIVYDRHAKPVASHQLEFKQHYPEAGWVEHDPMEIMESVKICMAKALDKAA
ADGHNMDVGLKAIGITNQRETTVMWSKSTGLPLYNAIVWMDARTSPICRRLESDLSGGRTHFVETCGLPI
STYFSALKVLWLIENVDAVKNAVRAGDALFGTIDTWLIWNLTGGIGGTDRDGNKVFGHHVTDCSNASRT
MLMNLKALDWDKPTLETLGIPAEILPKIISNSERIGVVANGFPLAGVPIAGCLGDQHAAMLGQLCQKGEA
KSTYGTGAFILLNTGEEPTQSSHGLLSTIAYKLGPSAPTNYALEGSIAIAGAAVQWLRDSLGIISTAADIEKL
ADTVQDSGGIYFVPAFNGLFAPWWRDDARGICIGITRFTNKGHIARAVLESMCFQVNDVLSSMHKDAGEA
GEVKSAEGEFLLRVDGGATVNNLLMQIQADLLGSPVVRPADIETTALGAAYAAGLAVGVWSKEQIFAGLH
KENTRVFRPKLDEAHRRKRADSWYKAVSRSFDLADLSL*
>AtNHO1 ABK32113.1
MAKENGFIGSIDQGTTSTRFIIYDHDARPVASHQVEFTQFYPEAGWVEHDPMEILESVKVCIAKALDKATA
DGHNVDGGLKAIGLTDQRETTVVWSKSTGLPLHKAIVWMDARTSSICRRLEKELSGGRSHFVESCGLPIST
YFSAMKLLWLMENVDDVKDAIKKGDAIFGTIDTWLIWNMTGGINGGLHVTDVTNASRTMLMNLKTLSW
DQDTLKTLGIPAEILPKIVSNSEVIGEICKGWPIPGIKIAGCLGDQHAAMLGQACRKGEAKSTYGTGAFILL
NTGEVPIKSGHGLLTTLAYKLGPQAQTNYALEGSIAIAGAAVQWLRDSLGIIKSASEIEDLAAMVDSTGGV
YFVPAFNGLFAPWWREDARGVCIGITRFTNKSHIARAVLESMCFQVKDVLDSMNKDAGEKGSLNNGKGE
FLLRVDGGATANNLLMQIQADLMGSPVVRPVDIETTALGAAYAARLAVGFWKEADIFESGEKAKNSKVFR
PAMEEGIRKKKVASWCKAVERTFDLADLSI*
>ZmGK NP_001132106.2
MAGKGKEVYVAAIDQGTTSTRFIVYDRHAKPVASHQLEFKQHYPEAGWVEHDPMEIIETVKVCMKEAVG
KAKDGKHNVVAGLKAIGITNQRETTVMWSKSTGRPLYNAIVWMDARTSPVCRRLENELSGGRTHFVETC
GLPISTYFSALKLLWLMENVDAVKDAVRTGDALFGTIDTWLIWNLTGGVAGGQHVTDCSNASRTMLMNL
KTLDWDKPTLAVLGVPVEILPKIISNSEKIGVVAKEFPFAGVPISGCLGDQHAAMLGQLCQKGEAKSTYGT
GAFILLNTGEEPTQSSHGLLSTIAYKLGPAAPTNYALEGSIAIAGAAVQWLRDSLGIIQSAAEIEKLAETVPD
SGGVYFVPAFNGLFAPWWRDDARGICIGITRFTNKGHIARAVLESMCFQVNDVLSSMHKDAGEAGEVKS
AEGEFLLRVDGGATVNNLLMQIQADLLGSPVVRPADIETTALGAAYAAGLAAGVWTKEKVFAGLHKENT
TVFRPKLDEAHRKKRADSWYKAVSRSFDLADLSL*
>SbGK XP_002448637.1
MAGEGEEVYVAAIDQGTTSTRFIVYDRHAKPVASHQLEFTQHYPEAGWVEHDPMEIIETVMVCMNEAVG
KAKDGKYNVVAGLKAIGITNQRETTVMWSKSTGHPLYNAIVWMDARTSPVCRRLESELSGGRTHFVERC
GLPISTYFSALKLLWLMENVDAVKDAIKTGDALFGTIDTWLIWNLTGGVAGGQHVTDCSNASRTMLMNL
KTLDWDKPTLDVLGVPVEILPKIISNSEKIGVVAKEFPFAGVPISGCLGDQHAAMLGQLCKKGEAKSTYGT
GAFILLNTGEEPTQSSHGLLSTIAYKLGPTAPTNYALEGSIAIAGAAVQWLRDSLGIIQTAAEIEKLAETVPDS
GGVYFVPAFNGLFAPWWRDDARGICIGITRFTNKGHIARAVLESMSFQVNDVLSSMHKDAGEAGEVKS
AEGEFLLRVDGGATVNNLLMQIQADLLGSPVVRPADIETTALGAAYAAGLAAGVWTKEQVFAGLHKENT
TVFRPKLDEAHRKKRADSWYKAVSRSFDLADLSL*
>PmGK RLM74845.1
MAGEGEEVYVAAIDQGTTSTRFIVYDRHAKPVASHQLEFKQHYPEAGWVEHDPMEIIETVKVCMKEAVD
NAKDGKYNVVAGLKAVGITNQRETTVMWSKSTGLPLYNAIVWMDARTSPVCRRLESELAGGRTHFVETC
GLPISTYFSAMKLLWLLENVDAVKDAVRTDDALFGTIDTWLIWNLTGGVHGGQHVTDCSNASRTMLMNL
KALDWDKPTLDALGIPAKILPKIISNSEKIGVVVDGFPLAGAPISGCLGDQHSAMLGQLCQKGEAKSTYGT
GAFILLNTGEEVTQSSHGLLSTIAYKLGPSAPTNYALEGSIAIAGAAVQWLRDSLGIIQTAAEIEKLAETVPD
SGGVYFVPAFNGLFAPWWRDDARGICIGITRFTNKGHIARAVLESMCFQVNDVLSSMHKDAGEAGEVKS

AEGDFLLRVDGGATVNNLLMQIQADLLGSPVVRPADIETTALGAAYAAGLAAGVWTKDQVFAGLHKDNT
TIFRPQLDETHRKKRADSWCKAVSRSFDLADLSL*

>AcGK XP_020090959.1

MAKEEAAAEVFVGSLDQGTTSTRFIIYDRNAKPIASHQVEFTQFYPEAGWVEHDPMEIMESVRVCMAKAI
DKATADGYNVDAGLKAVGLTNQRETTVAWSKSTGRPLYNAIVWMDVRTSSICRRLEKELSGGRNHFVDV
CGLPISTYFSAVKILWLMKNVDAVKSAVAAGDALFGTVDTWMIWNLTGGLHVTDCSNASRTMLMNIKTL
DWDKPTLETLGIPIEILPKIISNSELVGAIAKGWPLAGIPISGCLGDQHAAMLGQLCRKGEAKSTYGTGAFIL
LNTGEEIVRSSHGLLTTIAYKLGPSAPTNYAIEGSIAIAGAAVQWLRDGLGLIRSAGEIEELAESVENSGGVY
FVPAFNGLFAPWWRDDARGVCIGITRFTNKGHIARAVLESMCFQVNDVLNSMHKDVDEGEGEFLLRVDG
GATVNNLLMQIQADLLGSPVVRPADTETTALGAAYAAGLAIGLWTEEQIFAGEHKEKTTIFRPKLDEAERK
QRSECWFKAVSRTFDLADLSL*

>VvGK RVW92084.1

MAKEDVFVGSIDQGTTSTRFIIYDRSAQPVGSHQVEFTQFYPEAGWVEHDPMEILESVGVCIKKAIDKATA
DGHNVDSGLKAIGLTNQRETTLIWSKSTGLPLYHAIVWMDARTSSICRKLEKELPGGRTHFVETCGLPISTY
FSALKLLWLLENVDAVKKAVEAGDALFGTIDTWLIWNMTGGLNGGVHVTDVSNASRTMLMNLKTLDW
DKPTLDTLGISAEILPKIVSNAEIIGTVAKGWPIPGLPISGCLGDQHAAMLGQACRKGEAKSTYGTGAFILL
NTGEEVIESKHGLLTTLAFKLGREAPTNYALEGSIAIAGAAVQWLRDSLGIISSASEIEELAAKVDSSGGVYF
VPAFNGLFAPWWRDDARGVCIGITRFTNKSHIARAVLESMCFQVKDVLDSMHKDAGEKGEVKNEKGEFL
LRVDGGATINNLLMQIQADLLGNPVVRPADIETTALGAAYAAGLAVGIWTEDEIFDSGEKVKLATTFYPAL
DEERRNKKVESWCKAVSRTFDLADLSL*

>TaGK AGH08245.1

MAGNGKGEEVYVASIDQGTTSTRFIIYDRHASPVASHQLEFKQHYPEAGWVEHDPMEIIESVKVCMAKTL
EKAAANGLNVDAGLKAIGITNQRETTVVWSKSTGLPLYNAIVWMDVRTSSICRRLESELSGGRTHFVETC
GLPLSTYFSALKLLWLMENVDAVKDAVRAGDALFGTIDTWLIWNLTGGIGGKDRDGKELVGQHVTDCSN
AARTMLMNLKALDWDKPTLEALGIPAGILPKIISNSEKIGVVASGFPLAGVSISGCLGDQHAAMLGQLCQK
GEAKSTYGTGAFILLNTGEEVTQSTHGLLSTIAYKLGPDAPTNYALEGSIAIAGAAVQWLRDSLGIISSASEI
EGLAESVQDSGGIYFVPAFNGLFAPWWRDDARGICIGITRFTNKGHIARAVLESMCFQVNDVLNSMHKDA
GESGEVKSAEGEFLLRVDGGATINNLLMQIQADLLGSPVVRPADIETTALGAAYAAGLAAGVWTKEEIFAG
LHKENTTVFRPKLDDAHRKKRGDSWYKAVSRSFDLADLSL*

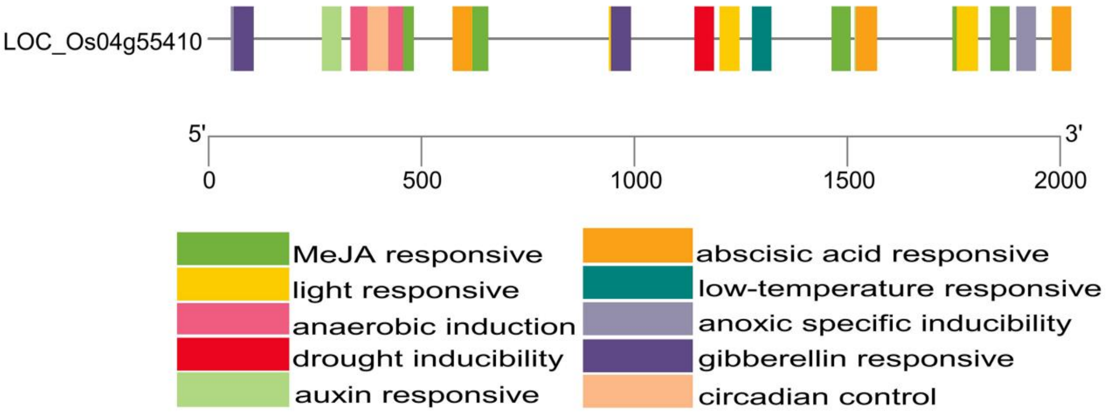

Figure S1 Visual analysis of promoter cis-acting elements using TBtools.

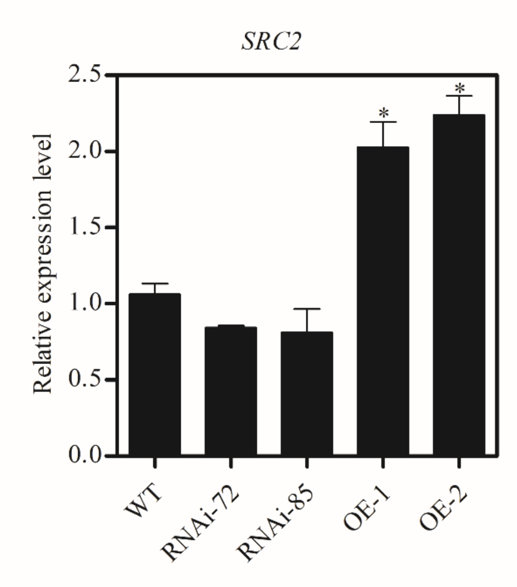

Figure S2 transcript levels of *OsSRC2* in *OsNHO1* transgenic plants
